# Accuracy of Patient Self-Report of Stroke: A Systematic Review from the UK Biobank Stroke Outcomes Group

**DOI:** 10.1371/journal.pone.0137538

**Published:** 2015-09-10

**Authors:** Rebecca Woodfield, UK Biobank Stroke Outcomes Group, UK Biobank Follow-up and Outcomes Working Group, Cathie L. M. Sudlow

**Affiliations:** 1 Clinical Centre for Brain Sciences, University of Edinburgh, Edinburgh, United Kingdom; 2 UK Biobank, Adswood, Stockport, United Kingdom; University of Glasgow, UNITED KINGDOM

## Abstract

**Objective:**

We performed a systematic review of the accuracy of patient self-report of stroke to inform approaches to ascertaining and confirming stroke cases in large prospective studies.

**Methods:**

We sought studies comparing patient self-report against a reference standard for stroke. We extracted data on survey method(s), response rates, participant characteristics, the reference standard used, and the positive predictive value (PPV) of self-report. Where possible we also calculated sensitivity, specificity, negative predictive value (NPV), and stroke prevalence. Study-level risk of bias was assessed using the Quality Assessment of Diagnostic Studies tool (QUADAS-2).

**Results:**

From >1500 identified articles, we included 17 studies. Most asked patients to report a lifetime history of stroke but a few limited recall time to ≤5 years. Some included questions for transient ischaemic attack (TIA) or stroke synonyms. No study was free of risk of bias in the QUADAS-2 assessment, the most frequent causes of bias being incomplete reference standard data, absence of blinding of adjudicators to self-report status, and participant response rates (<80%). PPV of self-report ranged from 22–87% (17 studies), sensitivity from 36–98% (10 studies), specificity from 96–99.6% (10 studies), and NPV from 88.2–99.9% (10 studies). PPV increased with stroke prevalence as expected. Among six studies with available relevant data, if confirmed TIAs were considered to be true rather than false positive strokes, PPV of self-report was >75% in all but one study. It was not possible to assess the influence of recall time or of the question(s) asked on PPV or sensitivity.

**Conclusions:**

Characteristics of the study population strongly influence self-report accuracy. In population-based studies with low stroke prevalence, a large proportion of self-reported strokes may be false positives. Self-report is therefore unlikely to be helpful for identifying cases without subsequent confirmation, but may be useful for case ascertainment in combination with other data sources.

## Introduction

Stroke is likely to be caused by a wide variety of genetic, lifestyle and environmental risk factors with individually modest effects and complex interactions [1, 2]. Very large studies, yielding large numbers of stroke outcomes, are required to investigate these effects reliably [3]. One such study is UK Biobank, a very large prospective cohort study of 503,000 participants, aged 40–69 years when recruited in England, Scotland and Wales between 2006 and 2010 [4]. At recruitment, participants completed a detailed touchscreen questionnaire which included self-report of previous medical conditions. Based on responses to the question ‘Has a doctor ever told you that you have had a stroke?’, the prevalence of stroke in UK Biobank estimated by self-report was 1.4% [5]. From published UK stroke prevalence data [6] and allowing for the healthy cohort effect (i.e., volunteers in population-based cohort studies tend to have lower disease rates on average than the general population), the true prevalence of stroke in UK Biobank is likely to be <2% (~5000 to 10,000 cases).

**Fig 1:**
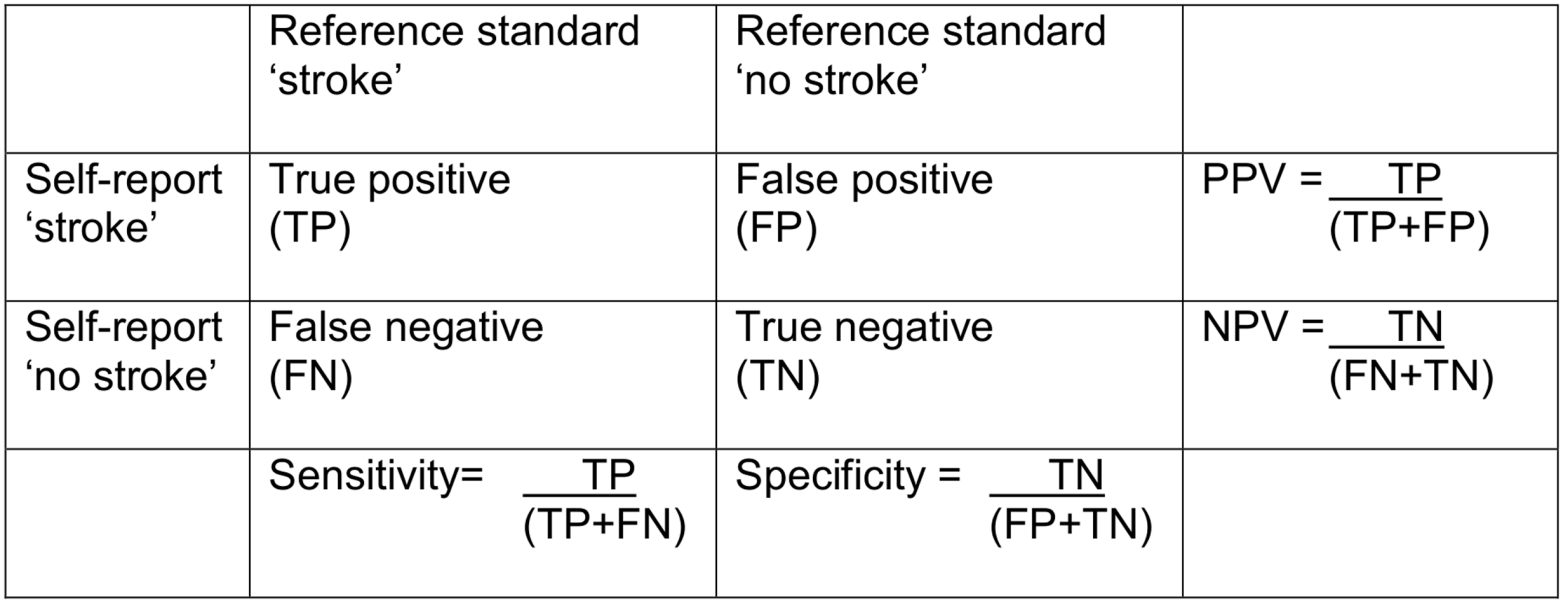
Calculation of PPV, sensitivity, specificity, NPV and stroke prevalence. PPV = positive predictive value; NPV = negative predictive value. Stroke prevalence = (TP+ FN) / (TP+FP+FN+TN).

For health-related outcomes such as stroke, large prospective studies such as UK Biobank need to maximise statistical power to detect genuine associations in nested case-control or case-cohort studies. This requires a strategy that identifies cases representative of the spectrum of the disease being studied with adequate sensitivity (the proportion of true positive stroke cases identified) and specificity (the proportion of true negative ‘non-stroke’ controls identified), and that maximises positive predictive value (PPV, the proportion of cases identified that are true cases of stroke) (Fig 1). PPV depends on sensitivity, specificity and stroke prevalence [7]. Maximising PPV will minimise the number of false positive cases, in turn minimising loss of statistical power through misclassification of cases. Some false negatives can be tolerated, since these are diluted by the very much larger control population, with much more limited impact on statistical power (in other words, negative predictive value and specificity will be always be very high where the condition being identified is uncommon).

Along with similar, large, prospective studies, UK Biobank aims to use multiple sources of data to identify stroke cases, including coded electronic data from hospital admissions, death certificates and primary care, as well as self-report. Use of multiple data sources should improve sensitivity of stroke detection, and, where multiple sources agree, improve PPV. However, potential cases, particularly those identified by a single data source, are likely to include false positives and may require further confirmation to maximise PPV.

Self-report accuracy varies according to the disease reported [8]. As far as we are aware, there are no systematic reviews of the accuracy of self-report of stroke. To assess the potential contribution of self-report to stroke identification methods in large prospective studies, we conducted a systematic review of published studies assessing the accuracy of patient self-report of stroke against a reference standard for stroke (using WHO [9] or equivalent definitions), focusing on PPV but also seeking information on sensitivity, specificity and negative predictive value (NPV).

## Methods

A detailed study protocol is available in S1 Appendix.

### Search Strategy

We searched Medline and Embase to November 2013 for studies assessing the accuracy of self-report of stroke against a reference standard diagnosis of stroke. We used a combination of medical subject heading and text word terms for ‘stroke’, ‘self-report’, ‘accuracy’, ‘medical records’ and ‘diagnosis’. We also searched the Cochrane Database of Systematic Reviews for relevant reviews of diagnostic test accuracy of stroke self-report. One author assessed eligibility by reviewing all titles and abstracts, and the full text of potentially relevant articles, resolving any uncertainties through discussion with a second. Bibliographies of included publications were reviewed to identify any additional relevant studies.

### Eligibility Criteria

We included studies which assessed the accuracy of patient self-report of stroke (with or without transient ischemic attack [TIA] or synonyms for either) against a reference standard diagnosis of stroke. We included studies which compared self-report of stroke or TIA against a reference standard of stroke because we hypothesised that asking about TIA (or its synonyms) might increase sensitivity for stroke. Included studies had to report the method of self-report, the reference standard used (any combination of hospital/primary care medical record review, hospital/primary care physician questionnaire, expert clinical examination, or hospital/population-based stroke registers), and the positive predictive value (PPV) +/- sensitivity, specificity, negative predictive value (NPV) of self-report (or provide data from which these values could be calculated, as shown in Fig 1). We excluded studies which assessed self-report of ‘cerebrovascular disease’, ‘symptoms’ or ‘past medical history’ unless stroke was specifically mentioned. We also excluded studies which used only coded data (e.g., International Classification of Diseases codes) as the reference standard to confirm cases, studies which did not distinguish confirmed stroke cases from transient ischaemic attack (TIA) or other cerebrovascular disease, and, to improve precision, studies with <50 self-reported strokes.

### Data Extraction

We extracted information from each included study on: the nature of the population surveyed (country, age range, selection criteria); number of participants included and response rate (proportion of potential participants who agreed to take part and completed questionnaires or attended interviews); question(s) asked (stroke, or stroke plus TIA/synonyms); mean (or median) age at self-report; recall period (years or lifetime); reference standard(s) used and source of data (‘hospital’ which includes only hospital diagnosed strokes, or ‘population’ which includes strokes diagnosed in the community); presence or absence of missing data; presence or absence of blinding of adjudicators (physicians or researchers who established the reference standard diagnosis) to participant self-report; presence or absence of differential verification (use of different reference standards for self-report positive versus self-report negative responses); PPV, sensitivity, specificity and NPV of self-report; number of reports of stroke which were confirmed TIAs.

### Data Analysis

We tabulated results for visual inspection to assess factors which might influence the accuracy of self-report including: estimated stroke prevalence; age at self-report (mean or median); recall time (years); question asked (stroke or stroke plus TIA/synonyms). Where possible, we used within-study comparisons to assess the influence of age, recall time and question(s) asked on the accuracy of self-report. The denominator population was the final number of participants (excluding non-responders) for whom complete reference standard data were available. Stroke prevalence was the number of ‘true strokes’ (TP+FN) divided by the denominator population (TP+FP+FN+TN) (Fig 1). We only calculated sensitivity, specificity and stroke prevalence when the reference standard was population-based (i.e., included general practitioner medical records, general practitioner questionnaires, or physician assessment of all participants, to capture strokes diagnosed out of hospital).

We assessed risk of bias at individual study level using the revised Quality Assessment of Diagnostic Studies tool (QUADAS-2),[10] but did not exclude studies on the basis of bias assessments. Risk of bias was scored as ‘low’, ‘high’, or ‘unclear’ in response to specific questions which considered patient selection (study design, sampling methods, exclusion criteria); index test (self-report questionnaire design, blinding to the reference standard); reference standard (source of data, blinding to self-report status); flow and timing (participant response rates, missing reference standard data, presence of differential verification). The study protocol (S1 Appendix) provides a detailed list of questions and scoring methods.

We calculated 95% confidence intervals for PPV, sensitivity, specificity, and NPV in *Stata* version 12 using the Wilson method for binomial proportions [11]. We did not undertake formal meta-analysis or meta-regression due to the heterogeneity between studies in their methods, participant characteristics and reporting, and because the number of studies available for inclusion in any potential meta-regression analysis was small (<10) [12].

## Results

**Fig 2:**
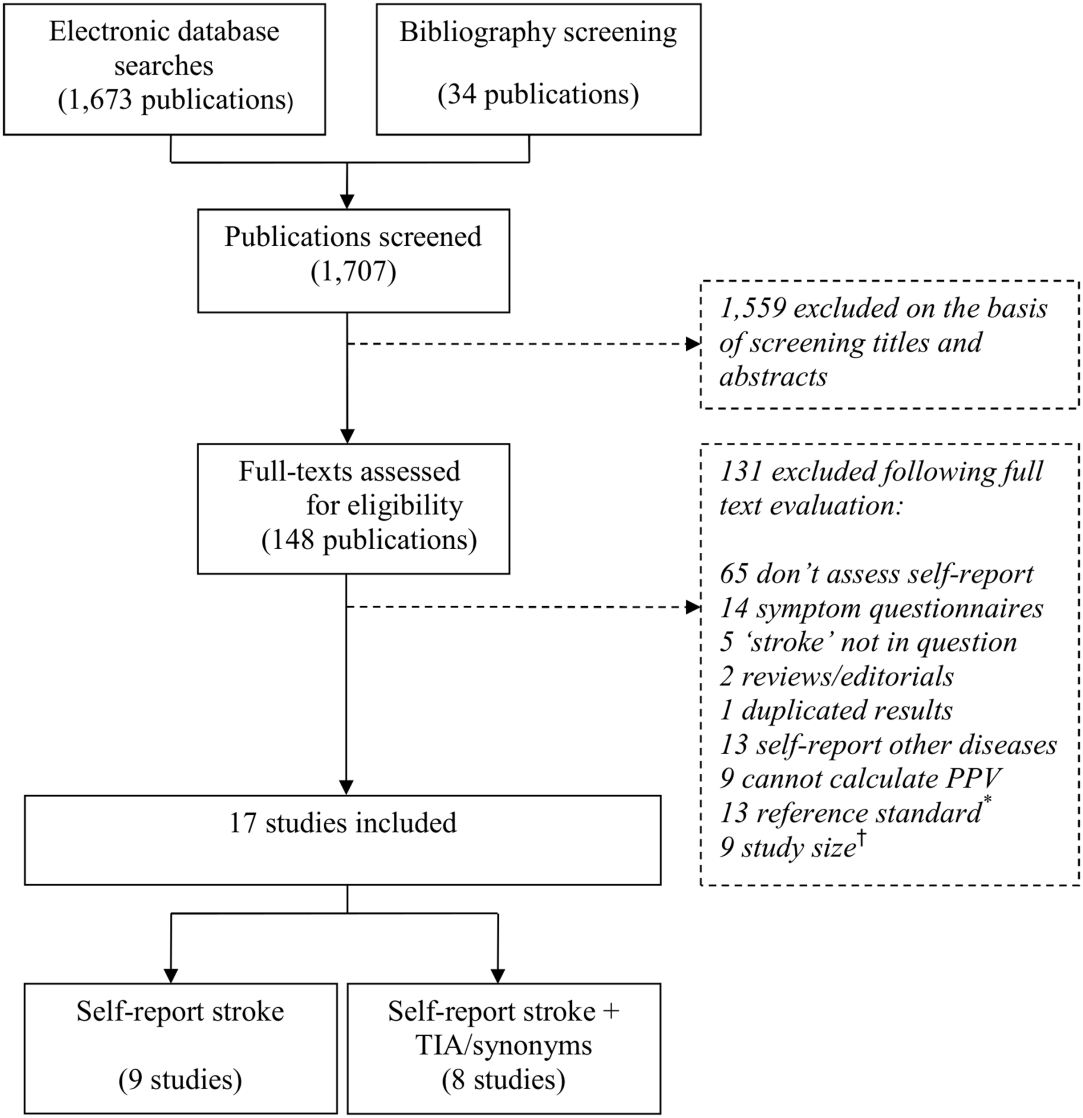
Study selection flow diagram. * Reference standard is cerebrovascular disease, or includes TIA, or uses ICD codes for stroke. ^†^ <50 self-reports of stroke validated, or number unpublished.

From 1707 publications identified, we reviewed 148 full texts, eventually including 17 studies (Fig 2).

### Characteristics of Included Studies

Characteristics of included studies are displayed in S1 Table. Studies were from the UK [13, 14, 15], elsewhere in Europe [16, 17, 18, 19, 20], Japan [21], North America [22, 23, 24, 25, 26, 27], Australia [28] and New Zealand [29]. Potential participants were selected based on geographic location and/or age in all studies. The age of potential participants ranged from >20 to >80 years old. Additional selection criteria in some studies included gender [13, 22, 24, 26], occupation [14, 24], presence or absence of disability [18, 22], absence of moderate/severe cognitive impairment [13, 22], place of residence [18], or medication use (non-steroidal anti-inflammatory drug prescription) [17].

Responses were ascertained by post [13–15, 17, 19, 21, 24–26], during routine outpatient visits [16], by face-to-face interview [18, 22, 23, 26–29], or by telephone [16]. Response rates were ≥80% in five studies [13, 15, 16, 18, 28], 60–79% in six [14, 20–23, 27], and <60% in three (23–57%) [17, 25, 29]. The remaining three studies did not report response rates [19, 24, 26]. Four studies compared characteristics of responders to non-responders [15, 17, 25, 28]. The two largest studies (including 10,000 and 120,000 potential participants) found that responders were older than non-responders and more often female [17, 28], but absolute differences were small (mean age 2 or 3 years higher, 1 or 6% more females).

Six studies asked participants to report ‘stroke’[13, 20, 22, 23, 28, 29], five to report ‘stroke, mini-stroke or transient ischemic attack (TIA)’ [14, 15, 25–27], and three to report stroke, but including stroke synonyms in the question (cerebral haemorrhage/brain haemorrhage/infarction/thrombosis/subarachnoid haemorrhage) [16, 19, 21]. All but three studies [17, 18, 24] published the specific question(s) asked. The period of recall ranged from six months to 5 years in five studies [14, 23, 24, 26, 28], 10 to 22 years in three studies [13, 21, 27], and was lifetime in the remaining nine [15–20, 22, 25, 29].

The reference standard was population-based (primary care data, primary care plus hospital data, and/or clinical examination of all participants) in thirteen studies [13–23, 25, 29], and hospital-based (hospital data only) in four [24, 26–28]. Stroke was confirmed by general practitioner questionnaire [17, 18], medical record review [16, 20, 24–28], presence on a stroke register plus medical record review [21], clinical examination plus medical record review [19], or a combination of these methods [13–15, 22, 23, 29]. Stroke prevalence ranged from 0.1%-17% in ten studies which used a population-based reference standard and published sufficient data to estimate prevalence [13, 15–18, 21, 22, 23, 25, 29].

### Assessment of Bias

Detailed results of the bias assessment are displayed in S2 Table. All studies had ‘high’ or ‘unclear’ risk of bias in at least one category. Incomplete reference standard data (missing or irretrievable records) [13, 15, 17–20, 22–24, 27–29], absence of blinding of adjudicators to self-report status [13–15, 19, 21, 23], and participant response rates (<80%) [14, 17, 20–23, 25, 27, 29] were the most frequent reasons for ‘high risk’ of bias.

Only five studies reported that adjudicators were blind to participant self-report results [17, 18, 24, 28, 29]. In six, presence or absence of blinding was not clearly reported [16, 20, 22, 25–27]. In one study, the reference standard diagnosis was made following physician examination of patients, un-blinded to self-report status [23]. In five other studies blinding was jeopardised because the reference standard included history and examination of patients [15, 19], or because records of apparent false-positive reports were re-examined for evidence of stroke [13, 14, 21]. In one of these studies, re-examination of records of apparent false-positive reports led to confirmation of a few additional stroke cases, and increased the PPV of patient self-report from 41% (95% CI 35–48) to 56% (95% CI 49–62).

The self-report method most often scored ‘unclear’ risk of bias. Three studies did not publish the specific question(s) asked [17, 18, 24], and eight (which used face-to-face interviews) did not report presence or absence of blinding of the interviewer to the reference standard diagnosis [16, 22, 23, 26–29].

Other sources of bias included use of hospital versus population-based reference standards [24, 26–28], exclusion of particular types of participants (eg., based on cognitive impairment, severe disability, or residence in a nursing home) [13, 18, 22, 23], and differential verification of the reference standard (different data used to verify self-report positive versus negative cases) [13–15, 21]. Most studies used a source of data (primary care records, general practitioner questionnaire, examination of all participants) which captured strokes diagnosed out of hospital [13–23, 25, 29]. The remaining studies, which used hospital-based reference standards [24, 26–28], had a higher risk of bias due to the potential for missing ‘true’ stroke cases diagnosed out of hospital.

### Accuracy of Self-Report

PPV of self-report ranged from 22–87%. Among ten studies which used a population-based reference standard, and had sufficient published data, sensitivity of self-report varied (from 36–98%), but specificity and NPV were consistently high (from 88–99.9%) (S3 Table).[15–18, 21–23, 25, 29] The proportion of self-reported strokes which were not strokes but confirmed to be TIAs (according to the reference standard) ranged from 6–25% among six studies with available relevant data [13, 15, 16, 20, 24, 29]. In these studies, if these confirmed TIAs were considered to be true rather than false positive stroke cases, the revised PPV was >75% in all but one study (Table 1).

**Table 1:** The proportion of self-reported strokes which were true stroke, true TIA or either.*

Study^†^	Country	Question asked^‡^	Self-report (n)	PPV True stroke (% & 95% CI)	PPV True TIA (% & 95% CI)	Revised PPV True stroke or TIA (% & 95% CI)
Machon	Spain	Stroke	176	22 (17–29)	6 (4–11)	28 (22–35)
Walker	UK	Stroke	201	56 (49–62)	25 (20–32)	81 (71–86)
O’Mahony	UK	Stroke/TIA	173	63 (56–70)	15 (11–22)	78 (71–84)
Engstad	Norway	Stroke	269	79 (74–84)	8 (4–10)	87 (82–90)
Bots	Netherlands	Stroke	285	67 (61–72)	10 (7–14)	77 (72–81)
Colditz	US	Stroke	115	66 (51–74)	21 (14–29)	87 (80–92)

TIA = Transient ischemic attack.

*According to the reference standard.

^†^Studies which published sufficient data (out of 17 included studies).

^‡^Participants were asked to report stroke, or stroke plus transient ischemic attack (TIA) (+/- synonyms for either).

### Factors Influencing Accuracy

The range of PPVs for self-report was similar [17, 25, 29] in the studies with very low response rates (<60%) to that of studies with higher response rates (≥60%) [13, 15, 16, 18, 19, 21, 27]. Overall, the range of PPVs appeared similar in blinded [17–19, 24, 28] versus un-blinded studies [13, 23] and in population-based [15–23, 25, 29] versus hospital-based [24, 26–28] studies.

#### Stroke prevalence

**Fig 3:**
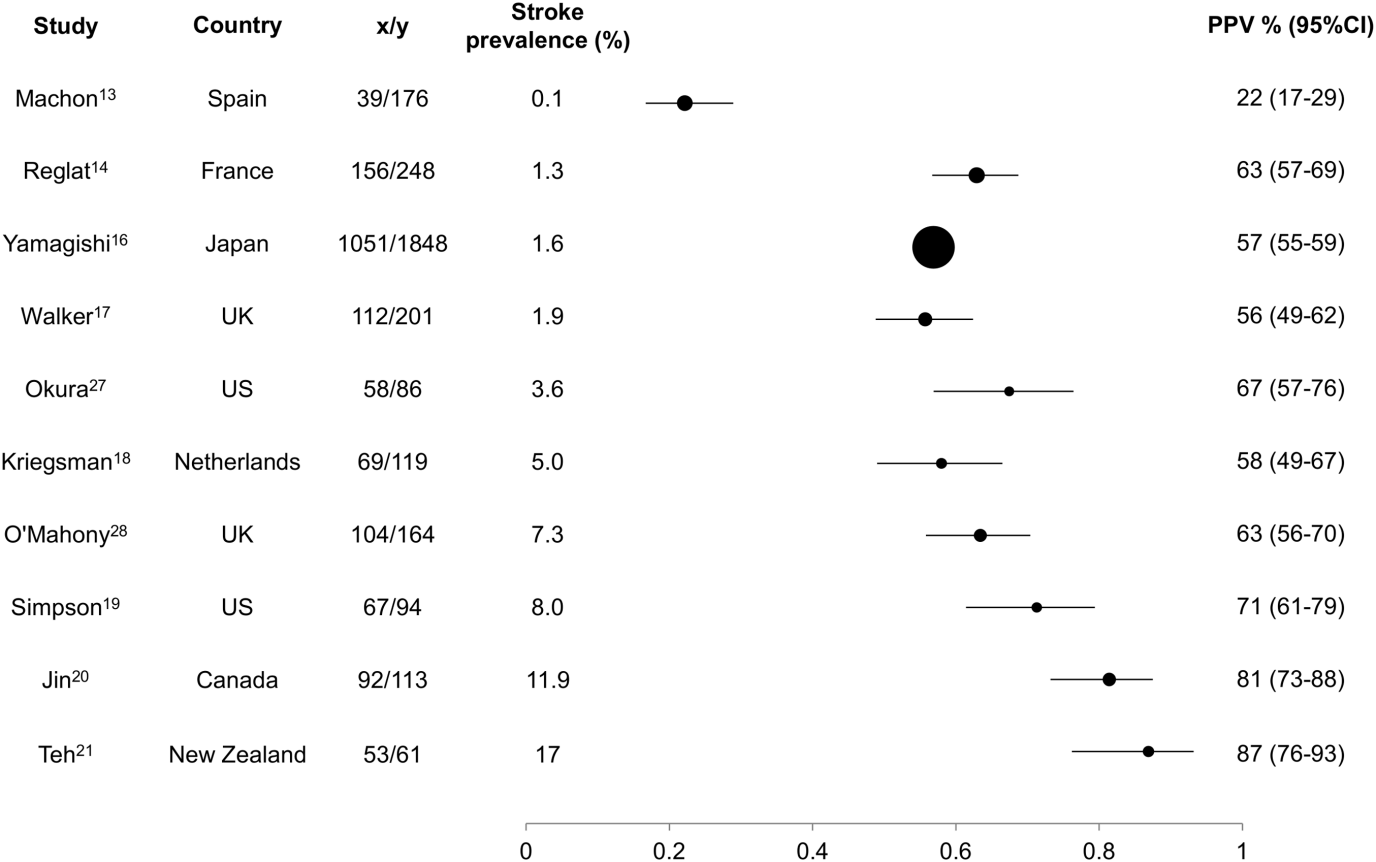
PPV of included studies with data on stroke prevalence. x/y: x = number of self-reported strokes which are confirmed; y = total number of self-reported strokes.

PPV increased with increasing stroke prevalence (Fig 3).

#### Participant age

Among studies which reported the average age of responders (mean or median) [15, 17, 19, 23, 25, 28, 29], we noted increasing PPV with increasing reporting age, probably because stroke prevalence increased with age (Table 2). Among five studies which published reporting age, and had sufficient data to calculate sensitivity [15, 17, 23, 25, 29], the study with the highest mean participant age (84 years) had the lowest sensitivity for stroke (sensitivity 36%, 95% CI 28–44) [29]. Sensitivity of self-report was stratified by age within one large study (~90,000 participants) [21], and fell with increasing age (78% in those <75 years versus 69% in those ≥75 years). A similar pattern was observed in a second, smaller study (~1, 536 participants. with sensitivities of 60% in those < 75 years versus 48% in those ≥ 75 years) [23]. However, limited data for sensitivity as well as heterogeneity between studies in population characteristics meant that it was not possible to demonstrate a clear association between reporting age and sensitivity.

**Table 2:** The influence of age on PPV, sensitivity, specificity and NPV of self-report.

Study*	Self-report (n)	Stroke prevalence (%)	Age† (mean)	PPV (% & 95%CI)	Sensitivity (% & 95%CI)	Specificity (% & 95%CI)	NPV (% & 95%CI)
Barr	87	-	52	38 (29–48)	-	-	-
Reglat	248	1.34	57	63 (57–69)	69 (62–74)	99 (99.3–99.6)	99.6 (99.5–99.7)
Engstad^‡^	269	-	60	79 (74–84)	-	-	-
Okura^§^	86	3.6	61^¶^	67 (57–76)	78 (68–86)	98.6 (97.9–99.0)	98.6 (97.9–99.0)
O'Mahony^§^	164	7	63	63 (56–70)	95 (89–98)	96 (94.5–96.7)	99.6 (99.0–99.8)
Jin	113	11.9	80	82 (73–88)	50 (43–57)	98 (97.6–98.9)	93.5 (92.1–94.7)
Teh	61	17	84	87 (76–93)	36 (28–44)	99 (97.7–99.3)	88.2 (85.8–90.3)

*Studies which published participant age at self-report (out of 17 included studies).

^†^Age at time of reporting

^‡^This study asked about stroke/synonyms

^§^These studies asked about stroke/TIA

^¶^Median

#### Question(s) asked

Overall, there was no clear difference in PPV or sensitivity between studies which asked about ‘stroke’ versus ‘stroke plus synonyms’ versus ‘stroke/TIA’ [13–16, 18, 19, 21–23, 25–27, 29]. However, among included studies there were no within-study comparisons of the influence of the question(s) asked on PPV or sensitivity of stroke self-report.

#### Recall time

In between-study comparisons, recall of events over the last six months to one year (PPV 72% to 81%) [23, 26] was not clearly more accurate than recall of events over the previous 2 to 5 years (PPV 38% to 78%) [14, 24, 28] or over lifetime (PPV 22% to 87%) [16–20, 22, 25, 29]. Two studies stratified PPV results by recall time, and neither found a difference in PPV for more versus less recent events [21, 28]. One of these studies (~ 90,000 participants) found no difference in sensitivity for more versus less recent events [21].

## Discussion

As far as we are aware this is the first systematic review of the accuracy of self-report of stroke. Self-report had variable PPV (range 22 to 87%) and variable sensitivity (range 36 to 98%) for stroke, but consistently high specificity and NPV (88 to 99%). In populations with low stroke prevalence, it would take a very large number of false positives to reduce specificity or NPV. PPV and sensitivity are therefore more informative measures. PPV increased with increasing stroke prevalence. Although this relationship is not surprising, we have shown that in populations with low stroke prevalence (<10%), a large proportion of self-reported strokes (~1/3 to 3/4) were false positive. This has important implications for large prospective studies, where stroke prevalence is likely to be low.

Reviews of the accuracy of self-report of various diseases have found that PPV and sensitivity vary depending on the disease reported [8]. For certain diseases, such as myocardial infarction, or cancer, a large proportion of false positive self-reports occur because patients confuse the diagnosis with a similar condition (e.g., other cardiovascular disease, or other cancer type) [30]. Similarly, we found that 6–25% of individuals who self-reported stroke had a reference standard diagnosis of TIA. If doctors or other health professionals used the term ‘mini-stroke’ when referring to TIA, the patient may be misled into thinking they had had a stroke. Grouping of stroke and TIA cases might be acceptable for some research questions (eg., those which explore common risk factors for stroke/TIA). If both TIA and stroke were considered true positive, the PPV of self-report of stroke (or stroke/TIA) was higher.

Other research questions require accurate identification of stroke, and accurate exclusion of TIA cases, (eg., those which explore risk-factors associated with the different pathological types and sub-types of stroke). However, there is no ‘gold standard’ diagnosis for stroke. The classic ‘symptom-based’ definition of stroke relies on symptom duration (>24 hours) to distinguish stroke from TIA [9]. A newer ‘tissue-based’ definition has been proposed, which relies on the presence of brain infarction to diagnose stroke, irrespective of symptom duration (<24 hours) [31], but application of this rule reclassifies cases of ‘TIA with infarction’ as stroke. Although physicians (expert and non-expert) are inconsistent in diagnosing stroke using ‘symptom-based’ definitions [32] the ‘tissue-based’ definition is equally susceptible to inter-observer variation [33–35]. Accurate diagnosis of brain infarction depends on the choice and timing of imaging, and on reviewer expertise [33, 34]. The ‘tissue-based’ definition is therefore likely to be particularly susceptible to variation when applied across different settings (with different brain imaging resources) [35]. To maintain consistency, we excluded studies which used the ‘tissue-based’ definition from our review. However, as new definitions and diagnostic terms continue to emerge, this lack of consistency will contribute to the misreporting of stroke (as TIA and vice versa) by patients and their physicians.

Previous primary studies have assessed the influence of gender, cognitive impairment, education, and number of co-morbidities on the accuracy or reliability of stroke self-report, with variable and sometimes conflicting results [18, 19, 22, 23, 25, 27, 29]. However, it is difficult to draw overall conclusions because a range of different methods were used to analyse data and present results.

We observed a wide range in PPV and sensitivity of self-report, which is likely to reflect between-study heterogeneity in both population characteristics and study design.

Reassuringly, only a few studies had low response rates (<60%). While this may have introduced selection bias, the accuracy (range of PPVs) of self-report was not clearly affected by response rates. Neither was there any clear affect of incomplete blinding on the range of PPVs. The majority of included studies had missing reference standard data [13, 15, 17–20, 22–24, 27– 29]. Although this is an important source of potential bias, incomplete reporting meant that it was not possible to assess the influence of missing reference standard data on the PPV and/or sensitivity of self-report. The reference standard used (hospital versus population-based) was an additional potential source of bias. Studies which excluded cases diagnosed out of hospital from their reference standard had a higher chance of misclassifying ‘true stroke cases’ as ‘false positive’ self-reports, and so of underestimating PPV. However, we did not find a difference in the overall accuracy of self-report (PPV) between hospital-based and population-based studies.

Strengths of this study include our thorough search strategy, adherence to published guidelines for test accuracy reviews [36], and inclusion of all relevant studies of stroke self-report. Although we only searched two online databases, a strategy which may have missed potentially relevant articles, we augmented our search by screening bibliographies of all included publications. Bibliography screening may be the most effective method of identifying additional relevant articles in systematic reviews of test accuracy [37]. Additional strengths of our review include the exclusion of studies which failed to distinguish TIA from stroke in the reference standard, use of a single stroke definition (WHO) [9], and exclusion of studies which used coded data as the only source of stroke confirmation.

There were some limitations. First, variation in the accuracy and completeness of the reference standard may have contributed to between-study variation in PPV and sensitivity. To improve comparability between studies, we only calculated sensitivity, specificity, NPV, or stroke prevalence when the reference standard was population-based. This was possible in ten out of seventeen included studies. However, only two included studies used the most robust population-based reference standard for stroke,[38], with multiple sources of case ascertainment and confirmation [14, 15]. Second, the true sensitivity of self-report is likely to be lower than the included studies suggest, since non-responders could not be included in the denominator population (non-response ranged from 10–77% amongst included studies). Third, incomplete reporting and limited within-study comparisons of population characteristics (such as age, gender, education, cognitive impairment, comorbidities) made it difficult to assess the influence of these individual factors on self-report PPV or sensitivity. Fourth, as discussed above, there is no gold standard test to diagnose stroke or TIA. Lack of consistency in determining the ‘true’ diagnosis is likely to have contributed to the wide range of reported PPV, sensitivity and stroke prevalence.

Further work is needed to assess and compare multiple overlapping sources of stroke detection in large epidemiological studies. Some studies have found that self-report increases the number of potential strokes detected (compared to hospital or primary care data) [14, 26]. However, it is uncertain whether using self-report is time- or cost-effective for stroke case detection in large prospective studies, because potential strokes would need to be confirmed, for example by medical record review. In addition, future work should examine the influence of the question asked on PPV and/or sensitivity of stroke report. We did not find a clear influence of the questions asked on PPV or sensitivity, but there were no within-study comparisons of stroke specific questions versus stroke/TIA or stroke synonyms. Establishing the best list of questions (to improve disease specific sensitivity or PPV) will be important for future questionnaire design. Future work could also consider the influence of new stroke definitions (where used), and more sensitive imaging methods (where available) on the PPV and/or sensitivity of self-report.

## Conclusions

Based on the results of this study, we suggest that self-report of stroke may be a useful screening tool to identify potential stroke disease in prospective studies, but not accurate enough on its own to confirm cases. Once potential cases are identified, a subsequent confirmation step using other data sources will also be required. The influence of stroke prevalence on PPV means that in studies with low stroke prevalence, like UK Biobank, a large proportion of potential strokes identified by self-report may be false positives.

## Supporting Information

S1 Appendix Study protocol. (DOCX)

S1 Table Characteristics of included studies. (DOCX)

S2 Table Assessment of bias. (DOCX)

S3 Table PPV, sensitivity and specificity of self-report (all included studies). (DOCX)

S4 Table PRISMA checklist. (DOC)
