# Supplementary material for: Accuracy of Patient Self-Report of Stroke: A Systematic Review from the UK Biobank Stroke Outcomes Group
Source: PLoS One. 2015 Sep 10;10(9):e0137538. doi: 10.1371/journal.pone.0137538 (PMC4565695; doi:10.1371/journal.pone.0137538)
Supplement: S1 Appendix — (DOCX) [file pone.0137538.s001.docx]

**S1 Appendix. Study Protocol.**

**Accuracy of patient self-report of stroke: a systematic review from the UK Biobank stroke outcomes group.**

**1. Review Question(s)**

1.1 Primary Question

Accuracy (Positive Predictive Value) of patient self-report of stroke (for example by self-administered questionnaire, or face-to-face interview) for a diagnosis of stroke (using WHO or equivalent definitions) in an adult population.

1.2 Secondary Questions

A. Sensitivity and specificity of patient self-report of stroke (amongst studies where the reference standard is population-based).

B. Influence of stroke prevalence on the accuracy (PPV) of self-report of stroke (amongst studies where the reference standard is population based).

C. Accuracy (Positive Predictive Value) of patient self-report of stroke for a diagnosis of Transient Ischaemic Attack (TIA).

D. Influence of the question(s) asked (for example stroke, or stroke plus TIA, or stroke plus TIA/synonyms for either) on the accuracy (PPV, sensitivity, specificity) of self-report of stroke.

E. Influence of period of recall (for example lifetime history versus more recent events) on the accuracy (PPV, sensitivity, specificity) of self-report of stroke.

F. Influence of participant age (mean, years) on the accuracy (PPV, sensitivity, specificity) of self-report of stroke.

**2. Searches**

We will search the following databases from inception to the date of search:

MEDLINE (Ovid SP);

EMBASE (Ovid SP)

Cochrane Register of Diagnostic Test Accuracy Studies

We will review bibliographies of included publications for any additional relevant articles.

2.1 MEDLINE search strategy

1. cerebrovascular disorders/ or exp basal ganglia cerebrovascular disease/ or exp brain ischemia/ or exp carotid artery diseases/ or exp cerebral small vessel diseases/ or exp intracranial arterial diseases/ or exp "intracranial embolism and thrombosis"/ or exp intracranial hemorrhages/ or stroke/ or exp brain infarction/ or stroke, lacunar/ or vertebral artery dissection/

2. (stroke or cerebrovasc$ or brain vasc$ or cerebral vasc$ or cva$ or apoplex$ or isch?emi$ attack$ or tia$ or SAH).tw.

3. ((brain$ or cerebr$ or cerebell$ or cortical or vertebrobasilar or hemispher$ or intracran$ or intracerebral or infratentorial or supratentorial or MCA or anterior circulation or posterior circulation or basal ganglia) adj5 (isch?emi$ or infarct$ or thrombo$ or emboli$)).tw.

4. ((brain$ or cerebr$ or cerebell$ or intracerebral or intracran$ or parenchymal or intraventricular or infratentorial or supratentorial or basal gangli$ or subarachnoid or putaminal or putamen or posterior fossa) adj5 (haemorrhage$ or hemorrhage$ or haematoma$ or hematoma$ or bleed$)).tw.

5. 1 or 2 or 3 or 4

6. questionnaires/ or self report/

7. self concept/ or self-assessment/ or self disclosure/ or diagnostic self evaluation/

8. Interviews as Topic/

9. Medical History Taking/

10. truth disclosure/

11. (self-report$ or questionnaire$).tw.

12. (patient$ adj5 report$).tw.

13. 6 or 7 or 8 or 9 or 10 or 11

14. "reproducibility of results"/ or "sensitivity and specificity"/ or "predictive value of tests"/

15. (positive predictive value or sensitivity or specificity).tw.

16. (agreement or validity or reliability or reproducibility or accuracy or accurate or concordance).tw.

17. 14 or 15 or 16

18. 5 and 13 and 17

19. records as topic/ or hospital records/ or exp medical records/ or nursing records/

20. exp Diagnosis/

21. ((diagnosis or diagnosed or history) adj5 stroke).tw.

22. ((hospital or GP or medical or general practitioner or health) adj5 (record or records)).tw.

23. cerebrovascular disorders/di or exp basal ganglia cerebrovascular disease/di or exp brain ischemia/di or exp carotid artery diseases/di or exp cerebral small vessel diseases/di or exp intracranial arterial diseases/di or exp "intracranial embolism and thrombosis"/di or exp intracranial hemorrhages/di or stroke/di or exp brain infarction/di or stroke, lacunar/di or vertebral artery dissection/di

24. 19 or 20 or 21 or 22 or 23

25. 18 and 24

2.2 EMBASE search strategy

1. cerebrovascular disease/ or basal ganglion hemorrhage/ or exp brain hematoma/ or exp brain hemorrhage/ or exp brain infarction/ or exp brain ischemia/ or exp carotid artery disease/ or cerebral artery disease/ or cerebrovascular accident/ or exp occlusive cerebrovascular disease/ or exp stroke/

2. stroke unit/ or stroke patient/

3. (stroke or cerebrovasc$ or brain vasc$ or cerebral vasc$ or cva$ or apoplex$ or isch?emi$ attack$ or tia$ or SAH).tw.

4. ((brain$ or cerebr$ or cerebell$ or cortical or vertebrobasilar or hemispher$ or intracran$ or intracerebral or infratentorial or supratentorial or MCA or anterior circulation or posterior circulation or basal ganglia) adj5 (isch?emi$ or infarct$ or thrombo$ or emboli$)).tw.

5. ((brain$ or cerebr$ or cerebell$ or intracerebral or intracran$ or parenchymal or intraventricular or infratentorial or supratentorial or basal gangli$ or subarachnoid or putaminal or putamen or posterior fossa) adj5 (haemorrhage$ or hemorrhage$ or haematoma$ or hematoma$ or bleed$)).tw.

6. 1 or 2 or 3 or 4 or 5

7. self-report/ or exp questionnaire/ or exp interview/

8. self concept/ or self evaluation/ or self disclosure/

9. health assessment questionnaire/ or health perceptions questionnaire/ or interpersonal communication/

10. anamnesis/ or medical history/

11. (self-report$ or questionnaire$).tw.

12. (patient$ adj5 report$).tw.

13. 7 or 8 or 9 or 10 or 11 or 12

14. "sensitivity and specificity"/

15. exp validity/ or exp reliability/ or reproducability/ or accuracy/ or predictive value/

16. (positive predictive value or sensitivity or specificity).tw.

17. (agreement or validity or reliability or reproducibility or accuracy or accurate or concordance).tw.

18. 14 or 15 or 16 or 17

19. 6 and 13 and 18

20. medical record/ or medical record review/ or electronic medical record/

21. exp diagnosis/

22. ((diagnosis or diagnosed or history) adj5 stroke).tw.

23. cerebrovascular disease/di or basal ganglion hemorrhage/di or exp brain hematoma/di or exp brain hemorrhage/di or exp brain infarction/di or exp brain ischemia/di or exp carotid artery disease/di or cerebral artery disease/di or cerebrovascular accident/di or exp occlusive cerebrovascular disease/di or exp stroke/di

24. ((hospital or GP or medical or general practitioner or health) adj5 (record or records)).tw.

25. 20 or 21 or 22 or 23 or 24

26. 19 and 25

**3. Types of study to be included**

We will include studies in any adult population (cohort studies, case-control studies, or clinical trials) which compare the self-report of stroke against a reference standard diagnosis of stroke (WHO, or equivalent definitions).

Studies are required to report the Positive Predictive Value of participant self-report (or data from which this can be calculated).

Studies should use a reference standard of ‘stroke’ (distinguished from transient ischaemic attack or generalised cerebrovascular disease) when calculating PPV, sensitivity, and specificity.

We will exclude studies which assess < 50 self-reported strokes (due to limited precision).

3.1 Condition or domain being studied

***Index test***: Self-report of stroke, or ‘stroke plus TIA’, or ‘stroke plus TIA/synonyms for either’ by any method (questionnaire or interview).

Studies should include the term ‘stroke’ in their questionnaire or interview. This excludes studies which assess self-report of symptoms, ‘cerebrovascular disease’, or past medical history for stroke.

3.2 Participants/population

Any adult population.

We will not exclude studies based on participant selection criteria (eg., age, cardiovascular risk, education, cognitive impairment, disability).

We have specified secondary questions which will examine the influence of participant characteristics (eg., age and stroke prevalence) on the accuracy of self-report.

3.3 Interventions, exposures

***Reference standard***: We accept that the inter-observer reliability of stroke diagnosis is imperfect, even amongst experts. In the absence of a true ‘gold standard’ for stroke, we will include studies which use any of the following reference standards for stroke: clinical examination; physician questionnaire; medical record review (primary care and/or hospital records); stroke registers (informed by multiple overlapping data sources, ‘hot pursuit’, and expert medical record review).

To improve accuracy we will exclude studies which use coded data (eg., International Classification of Diseases codes) for the reference standard, unless other methods (above) are also used.

Studies should use a symptom-based definition (WHO, or equivalent) for diagnosing stroke.

3.4 Comparators/control

Not applicable

**4. Context**

The principle criterion is self-report of stroke. We aim to inform approaches to stroke ascertainment in large population-based studies.

Depending on the design of the study, the measure of interest could be self-report of prevalent (lifetime stroke) or incident (since recruitment) events. We have pre-specified questions to see if the accuracy of self-report varies by length of recall time.

In UK Biobank, participants were asked to self-report stroke by questionnaire, and answers were later confirmed during a brief nurse-led interview. We will include studies which assess self-report by either self-administered questionnaire or face-to-face interview.

**5. Outcomes**

5.1 Primary outcomes

We will calculate Positive Predictive Value (PPV) of self-report for stroke in all included studies using the available published data.

5.2 Secondary outcomes

In studies which use population-based reference standards, we will calculate Sensitivity, Specificity, PPV, and stroke prevalence using 2x2 contingency tables.

The reference standard will be grouped into hospital-based versus population-based according to the following definitions:

Population-based: a reference standard which identifies strokes diagnosed out of hospital, and therefore captures as many ‘true’ strokes in the participant population as possible; primary care medical records; general practitioner questionnaires; population-based stroke registers (informed by expert medical record review); physician assessment of all participants.

Hospital-based: a reference standard which only identifies hospitalised strokes; hospital medical records; hospital physician questionnaires; hospital based stroke registers (informed by expert hospital record review).

**6. Data extraction (selection and coding)**

We will extract data onto study-specific proforma.

6.1 Covariates of interest:

- Study author

- Country

- Population selection criteria (eg., random or consecutive sampling, inclusions/exclusion criteria)

- Self-report method (postal questionnaire, telephone interview, face-to-face interview)

- Question(s) asked

- Participant response rates (% population who agree to take part, complete questionnaires, and/or attend interviews).

- Participant age at self-report (mean, median or range).

- Length of recall time (years, or lifetime)

- Reference Standard used (eg., medical records, physician questionnaire and whether hospital or population-based)

- Number(s) included in final analysis (2x2 table, or true positive versus false positive self-reported strokes for PPV calculation).

**-** Number(s) excluded from analysis (eg., because of incomplete reference standard data).

- Blinding (to self-report status for determination of the reference standard diagnosis)

6.2 Risk of bias (quality) assessment

We will assess methodological quality using the revised Quality Assessment of Diagnostic Accuracy Studies tool (QUADAS-2).

We will focus on assessment of bias questions rather than generalizability.

6.3 Modified QUADAS-2 questions for assessment of bias:

***1) Patient selection***

***a) Sampling method: was consecutive or random sampling used?***

Low risk of bias: consecutive or random sampling was used. Unclear risk of bias: insufficient information published. High risk of bias: did not use consecutive or random sampling.

***b) Study design: was a case-control study design avoided?***

Low risk of bias: case-control design avoided. High risk of bias: case-control design used. Unclear risk of bias: insufficient information published.

***c) Population included: were inappropriate exclusions avoided?***

Low risk of bias: inappropriate exclusions were avoided. High-risk of bias: participants were excluded based on characteristics which might influence self-report accuracy (eg, education level, cognitive impairment, disability.) Unclear risk of bias: insufficient information published.

***2) Index test (self-report)***

***a) Interpreted blind: was the index test interpreted without knowledge of the reference standard diagnosis?***

Risk of bias depends on the degree of interpretation of the index test (self-report) and the presence/absence of blinding to the reference standard diagnosis. Low risk; a ‘yes/’no’ answer given by the participant in a self-administered questionnaire, irrespective of blinding; self-report by face-to-face interview and blinding present. Unclear risk: more than a ‘yes’/’no’ answer in a self-administered questionnaire and blinding not reported; a ‘yes’/’no’ answer in a face-to-face interview and blinding not reported; question(s) asked not published. High risk: more than a ‘yes’/’no’ answer in a face-to-face interview and blinding either not reported or not present; more than a ‘yes’/’no’ answer in a self-administered questionnaire and blinding not present; face-to-face interview by medically trained professional, irrespective of blinding.

***b) Was the threshold pre-specified?***

Selecting the test threshold to optimize sensitivity/specificity may lead to overestimation of test performance. Low risk of bias: the question(s) asked were published and the answers accepted as positive self-report (e.g., stroke/stroke plus TIA/stroke plus TIA plus synonyms for either) were specified at the outset. Unclear risk of bias: the question(s) asked were not published but it was specified at the outset which answers were accepted as positive self-report. High risk of bias: the question(s) asked were not published and it was not specified at the outset which answers were accepted as positive self-report.

***3) Reference standard***

***a) Source of data: is the reference standard likely to correctly classify the target condition?***

Low risk of bias: the reference standard was ‘population-based’ and included ‘true stroke’ cases diagnosed out of hospital. Unclear risk of bias: insufficient information. High risk of bias: the reference-standard was ‘hospital-based’ and excluded ‘true stroke’ cases diagnosed out of hospital.

***b) Blind to self-report: were reference standard results interpreted without knowledge of results of the index test?***

Low risk of bias: the reference standard diagnosis was made blind to self-report results. Unclear risk of bias: blinding not reported. High risk of bias: the reference standard diagnosis was made with knowledge of self-report results.

***4) Flow and timing***

***a) Participant response rates: were all patients included in the analysis?***

Low risk of bias: participant response rates >80%. High risk of bias: participant response rates <80%. Unclear risk of bias: participant response rates not reported.

***b) Missing data: did all patients receive a reference standard?***

Low risk of bias: reference standard data available for all responding participants. Unclear risk of bias: insufficient information published. High risk of bias: responding participants excluded from the final analysis due to missing reference standard data or because the final reference standard diagnosis was ‘unclear’.

***c) Differential verification: did all patients receive the same reference standard?***

Low risk of bias: the same reference standard was used for all participants. Unclear risk of bias: different reference standards were used but it was unclear if this differed by self-report status. High risk of bias: a different reference standard was used if participants self-reported stroke (vs. self-reported no-stroke).

***d) Was there an appropriate interval between the index test and the reference standard?***

This question was excluded from our study because the time interval between the index test (self-report) and reference standard (‘true’ diagnosis of stroke versus non-stroke) did not influence the accuracy of the original diagnosis (which was made historically). The time interval could influence the availability of reference standard data, but this aspect of bias was already captured in question 4b (above).

**7. Strategy for data synthesis**

We will cross classify self-reported strokes with the reference standard diagnosis (‘stroke’ versus ‘non-stroke’).

PPV (%) = true positive reports / [true positive reports + false positive reports] x 100

True positive reports = self-report ‘stroke’ and reference standard ‘stroke’.

False positive reports = self-report ‘stroke’ and reference standard ‘non-stroke’.

Where the reference standard is population based, we will construct standard 2x2 tables describing binary test results (self-report ‘stroke’ and self-report ‘non-stroke’) cross classified with binary reference standard results (‘stroke’ and ‘non-stroke’).

We will use this data to calculate sensitivity, specificity, PPV, and 95% confidence intervals. Stroke prevalence will be calculated using ‘total reference standard stroke’/ ‘final included population’.

We will tabulate results for visual inspection to assess the influence of participant age, question(s) asked, recall time, and stroke prevalence on the PPV, sensitivity and/or specificity of self-report. Where possible, and to limit the impact of between-study heterogeneity, we will use within-study as well as between-study comparisons.

We will assess heterogeneity between studies by inspection of tabulated data.

We will not quantify publication bias as there is no assessment applicable to test accuracy.

**8. Dissemination plans**

We will present our findings at local, national and international meetings. We plan to publish a full paper in a peer-reviewed scientific journal.
