# Supplementary material for: Accuracy of Patient Self-Report of Stroke: A Systematic Review from the UK Biobank Stroke Outcomes Group
Source: PLoS One. 2015 Sep 10;10(9):e0137538. doi: 10.1371/journal.pone.0137538 (PMC4565695; doi:10.1371/journal.pone.0137538)
Supplement: S1 Table — (DOCX) [file pone.0137538.s002.docx]

**S1 Table. Characteristics of included studies.**

|  | **Patient selection^*^** | | | **Index test^†^ (self-report)** | | | **Reference standard** | | **Flow and timing** | | |
| --- | --- | --- | --- | --- | --- | --- | --- | --- | --- | --- | --- |
| **Study** | **Country** | **Target population**  **age (range)** | **Population included** | **Self-report method** | **Question(s)**  **asked** | **Recall period**^‡^ | **Source of data**^§^ | **Blind to self-report ^¶^** | **Response rate^**^**  **(%)** | **Missing data^††^** | **Differential verification** |
| Reglat | France | > 20 | Prescribed  NSAIDs^‡‡^ | Postal survey | - | Lifetime | Population^§§^ | Yes | 23^¶^**^¶^** | Yes | No |
| Yamagishi | Japan | 40-69 | - | Postal survey | Stroke+3 | 10 | Population^***^ | No^***^ | 77 | No | Yes^***^ |
| Walker | UK | 40-59 | Men**^†††^** | Postal survey | Stroke | 12-14 | Population^‡‡‡^ | No^‡‡‡^ | 90 | Yes | Yes^‡‡‡^ |
| Kriegsman | Netherlands | 55-85 | Independent^§§§^ | Interview | - | Lifetime | Population^§§^ | Yes | 82 | Yes | No |
| Simpson | US | > 65 | Disabled women^¶¶¶^ | Interview | Stroke | Lifetime | Population | Unclear | 71 | Yes | Unclear |
| Jin | Canada | > 65 | Cognitive impairment**^****^** | Interview | Stroke | 1 | Population | No | 64 | Yes | Unclear |
| Engstad | Norway | > 24 | - | Postal survey | Stroke+1 | Lifetime | Population | No^††††^ | Unclear | Yes | No |
| Barr | Australia | >25 | - | Interview | Stroke | 5 | Hospital | Yes | 82 | Yes | No |
| Bots | Netherlands | > 55 | - | - | Stroke | Lifetime | Population | Unclear | 78 | Yes | Unclear |
| Britton | UK | 35 -55 | Civil servants | Postal survey | Stroke/TIA | 3 - 4 | Population^‡‡‡‡^ | No^‡‡‡‡^ | 66 - 70 | No | Yes^‡‡‡‡^ |
| Colditz | US | 30-55 | Female nurses | Postal survey | - | 2 | Hospital | Yes | Unclear | Yes | No |
| Teh | New Zealand | >80 | - | Interview | Stroke | Lifetime | Population | Yes | 56 | Yes | No |
| Machon | Spain | 29-70 | - | Questionnaire/  Interview | Stroke+2 | Lifetime/3^§§§§^ | Population | Unclear | 99 | No | No |
| O’Mahony | UK | >45 | - | Postal survey | Stroke+5/TIA | Lifetime | Population | No^¶¶¶¶^ | 83 | Yes | Yes^¶¶¶¶^ |
| Heckbert | US | 50-79 | Postmenopausal women ^*****^ | Interview/  Postal survey | Stroke/TIA | 0.5 - 1 | Hospital | Unclear | Unclear | Unclear | No |
| Okura | US | >45 | - | Postal survey | Stroke/TIA | Lifetime | Population | Unclear | 47 | No | No |
| Bergmann | US | 25-74 | - | Interview | Stroke/TIA | 12-22 | Hospital | Unclear | 78 | Yes | No |

Stroke/TIA: Has a doctor ever told you that you had a stroke, mini stroke or Transient Ischemic Attack (TIA)?

Stroke +1: Do you have or have you ever had a stroke or cerebral haemorrhage?

Stroke +2: Have you ever been told by a physician that you have or have had a stroke, cerebral thrombosis, or cerebral haemorrhage?

Stroke+3: Have you ever been told by a physician that you had a stroke, cerebral haemorrhage, cerebral infarction, or subarachnoid haemorrhage?

Stroke+5: Have you ever had a stroke, cerebral haemorrhage, cerebral thrombosis, brain haemorrhage, subarachnoid haemorrhage, or cerebrovascular accident?

^*^Apart from four studies which did not clearly report their sampling methods,^14,16,17,21^ all included studies randomly sampled participants from within their chosen sampling frame.

^†^None of the included studies reported if self-report was interpreted blind to the reference standard diagnosis.

^‡^Recall period for self-report of incident stroke. ‘Lifetime’ refers to self-report of stroke at any time prior to recruitment to a study.

^§^Source of reference standard data. Population: primary care medical records and/or general practitioner questionnaires and/or population-based stroke registers and/or clinical examination of all participants. Hospital: hospital-based records only +/- hospital physician questionnaire.

^¶^Yes: the reference standard diagnosis was made blind to patient self-report status. Unclear: not clearly reported. No: the reference standard diagnosis was made un-blind to self-report status, or blinding was jeopardised (history and examination of participants, or differential verification).

^**^ Proportion of potential participants who agreed to take part, completed and returned questionnaires, or attended interviews.

^††^ Yes: participants excluded from the final analysis due to missing reference standard data, or because the reference standard diagnosis was ‘unclear’. Unclear: insufficient information published. No: reference standard data complete for all participants.

^‡‡^Patients who had been prescribed Celecoxib, Rofecoxib, or traditional non-selective Non-Steroidal Anti-Inflammatory Drugs (NSAIDs) on a monthly basis for 1 year.

^§§^General Practitioner questionnaire only.

**^¶^**^¶^Amongst these participants ~58% of GPs responded with questionnaire data.

^***^Population stroke register. Medical records were re-examined if a patient reported stroke but the register was negative.

**^†††^**Excluded those with ‘severe mental or physical disability’.

^‡‡‡^General Practitioner questionnaire. Primary care records were re-examined for evidence of stroke if a patient reported stroke but the GP did not (apparent false positive cases). This led to confirmation of a few additional stroke cases, and increased the PPV of patient self-report from 41% (95% CI 35-48) to 56% (95% CI 49-62).

^§§§^Excluded residential or nursing home residents.

^¶¶¶^Excluded participants with moderate or severe cognitive impairment.

^****^The majority of participants with normal cognition were excluded. The final sample included participants with cognitive impairment and a random sample without cognitive impairment.

^††††^In this study, physicians took a history from patients. Results of history and physical examination were used, in addition to medical record review, to determine the final diagnosis (presence or absence of stroke).

^‡‡‡‡^ Source of the reference standard was coded hospital data plus medical record review or GP questionnaire. If self-report or coded data were positive for stroke, a committee reviewed data abstracted from medical records. If self-report was positive for stroke and hospital data were unavailable, GP questionnaire was requested.

^§§§§^Self-report included prevalent events (baseline questionnaire) plus additional prevalent or incident events (3 year follow-up telephone interview).

^¶¶¶¶^Validation included physician history and examination of patients who self-reported stroke. The final reference standard diagnosis was made by expert consensus using multiple sources of information. Good medical records took precedence over the physician diagnosis at home visit.

^*****^Includes clinical trial participants on different HRT regimens, low fat diet, and calcium and vitamin D supplementation.
