# Supplementary material for: Accuracy of Patient Self-Report of Stroke: A Systematic Review from the UK Biobank Stroke Outcomes Group
Source: PLoS One. 2015 Sep 10;10(9):e0137538. doi: 10.1371/journal.pone.0137538 (PMC4565695; doi:10.1371/journal.pone.0137538)
Supplement: S2 Table — (DOCX) [file pone.0137538.s003.docx]

**S2 Table. Study-level risk of bias using the Quality Assessment of Diagnostic Studies tool (QUADAS-2).**

| **STUDY** | **PATIENT SELECTION** | | | **INDEX TEST** | | **REFERENCE STANDARD** | | **FLOW AND TIMING** | | |
| --- | --- | --- | --- | --- | --- | --- | --- | --- | --- | --- |
|  | **Sampling method^*^** | **Study design^†^** | **Population included**^‡^ | **Interpreted blind^§^** | **Threshold specified^¶^** | **Source^**^** | **Blind to self-report^††^** | **Response rate^‡‡^** | **Missing data^§§^** | **Differential verification^¶¶^** |
| Reglat | ☺ | ☺ | ☺ | ? | ? | ☺ | ☺ | ☹ | ☹ | ☺ |
| Yamagishi | ☺ | ☺ | ☺ | ☺ | ☺ | ☺ | ☹ | ☹ | ☺ | ☹ |
| Walker | ☺ | ☺ | ☹ | ☺ | ☺ | ☺ | ☹ | ☺ | ☹ | ☹ |
| Kriegsman | ? | ☺ | ☹ | ☹ | ? | ☺ | ☺ | ☺ | ☹ | ☺ |
| Simpson | ☺ | ☺ | ☹ | ? | ☺ | ☺ | ? | ☹ | ☹ | ? |
| Jin | ? | ☺ | ☹ | ? | ☺ | ☺ | ☹ | ☹ | ☹ | ? |
| Engstad | ☺ | ☺ | ☺ | ☺ | ☺ | ☺ | ☹ | ? | ☹ | ☺ |
| Barr | ☺ | ☺ | ☺ | ? | ☺ | ☹ | ☺ | ☺ | ☹ | ☺ |
| Bots | ☺ | ☺ | ☺ | ? | ☺ | ☺ | ? | ☹ | ☹ | ? |
| Britton | ☺ | ☺ | ☺ | ☺ | ☺ | ☺ | ☹ | ☹ | ☺ | ☹ |
| Colditz | ? | ☺ | ☺ | ? | ? | ☹ | ☺ | ? | ☹ | ☺ |
| Teh | ? | ☺ | ☺ | ? | ☺ | ☺ | ☺ | ☹ | ☹ | ☺ |
| Machon | ☺ | ☺ | ☺ | ? | ☺ | ☺ | ? | ☺ | ☺ | ☺ |
| O’Mahony | ☺ | ☺ | ☺ | ☺ | ☺ | ☺ | ☹ | ☺ | ☹ | ☹ |
| Heckbert | ☺ | ☺ | ☺ | ? | ☺ | ☹ | ? | ? | ? | ☺ |
| Okura | ☺ | ☺ | ☺ | ☺ | ☺ | ☺ | ? | ☹ | ☺ | ☺ |
| Bergman | ☺ | ☺ | ☺ | ? | ☺ | ☹ | ? | ☹ | ☹ | ☺ |

☺Low risk of bias ☹High risk of bias ? Unclear risk of bias

Rules for assessment of bias are displayed in S1 Appendix.

^*^Was consecutive or random sampling used?

^†^Was a case-control design avoided?

^‡^Were inappropriate exclusions avoided?

^§^Was self-report interpreted blind to the reference standard diagnosis?

^¶^Was the test threshold (self-report positive versus self-report negative) pre-specified?

^**^Is the reference standard likely to correctly classify the target condition?

**^††^**Was the reference standard diagnosis made blind to participant self-report status?

^‡‡^Were all participants included in the analysis?

^§§^Did all participants receive a reference standard?

^¶¶^Did all participants receive the same reference standard?
