# Supplementary material for: Accuracy of Patient Self-Report of Stroke: A Systematic Review from the UK Biobank Stroke Outcomes Group
Source: PLoS One. 2015 Sep 10;10(9):e0137538. doi: 10.1371/journal.pone.0137538 (PMC4565695; doi:10.1371/journal.pone.0137538)
Supplement: S3 Table — (DOCX) [file pone.0137538.s004.docx]

**S3 Table. PPV, sensitivity, specificity and NPV of self-report^*^ (all included studies).**

| **Study** | **Study population^†^**  **TP+FP+FN+TN** | **Self-report**  **stroke**  **TP+FP** | **Self-report confirmed**  **TP** | **Total**  **stroke**  **TP+FN** | **Stroke prevalence (%)**  **(TP+FN)/(TP+FP+FN+TN)** | **PPV**  **(% & 95% CI)**  **TP/(TP+FP)** | **Sensitivity**  **(% & 95% CI)**  **TP/(TP+FN)** | **Specificity**  **(% & 95% CI)**  **TN/(FP+TN)** | **NPV**  **(% & 95%CI)**  **TN/(TN+FN)** |
| --- | --- | --- | --- | --- | --- | --- | --- | --- | --- |
| Reglat | 16935 | 248 | 156 | 227 | 1.3 | 63 (57-69) | 69 (62-74) | 99 (99.3-99.6) | 99.6 (99.5-99.7) |
| Yamagishi | 89914 | 1848 | 1051 | 1447 | 1.6 | 57 (55-59) | 73 (70-75) | 99.1 (99.0-99.2) | 99.5 (99.5-99.6) |
| Walker | 5907 | 201 | 112 | 126 | 1.9 | 56 (49-62) | 89 (82-93) | 97 (96.8-97.7) | 99.8 (99.6-99.9) |
| Kriegsman | 2380 | 119^‡^ | 69^‡^ | 119^‡^ | 5.0 | 58 (49-66) | 58 (49-66) | 98^‡^ (97.1-98.3) | 97.8 (97.1-98.3) |
| Simpson | 945 | 94 | 67 | 68 | 8.0 | 71 (61-79) | 98 (92-99) | 97 (95.7-97.9) | 96.9 (95.5-97.9) |
| Jin | 1536 | 113 | 92 | 184 | 11.9 | 81 (73-88) | 50 (43-57) | 98 (97.6-98.9) | 93.5 (92.1-94.7) |
| Engstad^§^ | 17122^§^ | 269 | 213 | -^§^ | -^§^ | 79 (74-84) | -^§^ | -^§^ | -^§^ |
| Barr | - | 87 | 33 | ^-^ | ^-^ | 38 (29-48) | ^-^ | ^-^ | ^-^ |
| Bots^¶^ | -^¶^ | 285 | 191 | -^¶^ | -^¶^ | 67 (61-72) | -^¶^ | -^¶^ | -^¶^ |
| Britton^¶^ | -^¶^ | 106 | 83 | -^¶^ | -^¶^ | 78 (70-85) | -^¶^ | -^¶^ | -^¶^ |
| Colditz | - | 115 | 76 | - | - | 66 (57-74) | - | - | - |
| Teh | 876 | 61 | 53 | 149 | 17.0 | 87 (76-93) | 36 (28-44) | 99 (97.7-99.3) | 88.2 (85.8-90.3) |
| Machon | 33554 | 176 | 39 | 48 | 0.1 | 22 (17-29) | 81 (68-90) | 99.6 (99.5-99.7) | 99.9 (99.94-99.98) |
| O’Mahony | 1508 | 164 | 104 | 110 | 7.3 | 63 (56-70) | 95 (89-98) | 96 (94.5-96.7) | 99.6 (99.0-99.8) |
| Heckbert | - | 854 | 614 | - | - | 72 (69-75) | - | - | - |
| Okura | 2,037 | 86^**^ | 58^**^ | 74^**^ | 3.6 | 67 (57-76) | 78 (68-86) | 98.6 (97.9-99.0) | 98.6 (97.9-99.0) |
| Bergmann | - | 113 | 76 | - | ^-^ | 67 (58-75) | ^-^ | ^-^ | ^-^ |

^*^ Fig 1. Shows how PPV, sensitivity, specificity, and NPV were calculated.

^†^Unless otherwise stated, the study population is the number of participants (non-responders excluded) for whom reference standard data was available. If left blank, the study only compared participants who self-reported stroke (TP+FP) against the reference standard.

^‡^These values were published as percentages.

^§^It was not possible to calculate sensitivity, specificity, or NPV in this study because validation was performed in a very small selected sample of the self-report negative (‘no-stroke’) participants.

^¶^In these studies, although the source of reference standard data was population based (GP questionnaire included to capture strokes diagnosed out of hospital), only those participants who self-reported stroke (‘stroke positive’ participants), plus a very small proportion of self-report negative (‘no-stroke’) were validated. There was insufficient data published to calculate sensitivity, specificity, NPV, or stroke prevalence.

^**^Calculated using published PPV, sensitivity, and number of strokes confirmed by medical record review.
